# Priority target conditions for algorithms for monitoring children's growth: Interdisciplinary consensus

**DOI:** 10.1371/journal.pone.0176464

**Published:** 2017-04-27

**Authors:** Pauline Scherdel, Rachel Reynaud, Christine Pietrement, Jean-François Salaün, Marc Bellaïche, Michel Arnould, Bertrand Chevallier, Hugues Piloquet, Emmanuel Jobez, Jacques Cheymol, Emmanuelle Bichara, the EBGM III study group, Barbara Heude, Martin Chalumeau

**Affiliations:** 1 INSERM, UMR1153 Epidemiology and Biostatistics Sorbonne Paris Cité Center (CRESS), early ORigins of the Child’s Health and Development Team (ORCHaD), Paris Descartes University, Paris, France; 2 INSERM, UMR1153 Epidemiology and Biostatistics Sorbonne Paris Cité Center (CRESS), Obstetrical, Perinatal and Pediatric Epidemiology Research Team (EPOPé), Paris Descartes University, Paris, France; 3 Paris-South University, Paris, France; 4 Société Française d’Endocrinologie et Diabétologie Pédiatrique, Marseille, Centre de Recherche en Neurobiologie et Neurophysiologie de Marseille (CRN2M), Centre National de la Recherche Scientifique, UMR 7286, Aix-Marseille University, Marseille, France; 5 Société de Néphrologie Pédiatrique, Department of Pediatrics (Nephrology unit), University Hospital of Reims, Reims, Laboratory of Biochemistry and Molecular Biology, Faculty of Medicine, Reims, France; 6 Association Française de Pédiatrie Ambulatoire, Commission Recherche, Gradignan, Pediatric office, St-Brieuc, France; 7 Groupe Francophone d'Hépato-Gastroentérologie et Nutrition Pédiatriques and Department of Pediatric Gastroenterology and Nutrition, Robert-Debré hospital, AP-HP, Paris, France; 8 Société Française de Médecine Générale, Commission Recherche, Orléans, Institut de Chimie Organique et Analytique (ICOA), Orléans University, UMR-CNRS 7311, Orléans, France; 9 Groupe de Pédiatrie Générale - Société Française de Pédiatrie, Boulogne-Billancourt, Department of Pediatrics and Pediatric Emergency, Ambroise-Paré hospital, Boulogne-Billancourt, France; 10 Groupe Francophone d'Hépato-Gastroentérologie et Nutrition Pédiatriques, Nantes, Division of Pediatric Gastroenterology, hôpital Mère-Enfants, Nantes, France; 11 Société de Formation Thérapeutique du Généraliste, Commission Recherche, Paris, France; 12 Commission de Santé Publique et de Pédiatrie Sociale - Société Française de Pédiatrie, Clichy, France; 13 Department of General Pediatrics, Necker Children's Hospital, AP-HP, Paris Descartes University, Paris, France; Centre Hospitalier Universitaire Vaudois, FRANCE

## Abstract

**Background:**

Growth monitoring of apparently healthy children aims at early detection of serious conditions through the use of both clinical expertise and algorithms that define abnormal growth. Optimization of growth monitoring requires standardization of the definition of abnormal growth, and the selection of the priority target conditions is a prerequisite of such standardization.

**Objective:**

To obtain a consensus about the priority target conditions for algorithms monitoring children's growth.

**Methods:**

We applied a formal consensus method with a modified version of the RAND/UCLA method, based on three phases (preparatory, literature review, and rating), with the participation of expert advisory groups from the relevant professional medical societies (ranging from primary care providers to hospital subspecialists) as well as parent associations. We asked experts in the pilot (n = 11), reading (n = 8) and rating (n = 60) groups to complete the list of diagnostic classification of the *European Society for Paediatric Endocrinology* and then to select the conditions meeting the four predefined criteria of an ideal type of priority target condition.

**Results:**

Strong agreement was obtained for the 8 conditions selected by the experts among the 133 possible: celiac disease, Crohn disease, craniopharyngioma, juvenile nephronophthisis, Turner syndrome, growth hormone deficiency with pituitary stalk interruption syndrome, infantile cystinosis, and hypothalamic-optochiasmatic astrocytoma (in decreasing order of agreement).

**Conclusion:**

This national consensus can be used to evaluate the algorithms currently suggested for growth monitoring. The method used for this national consensus could be re-used to obtain an international consensus.

## Introduction

The objective of monitoring the growth of apparently healthy children is to detect serious conditions early [1]. This monitoring combines clinical expertise and the use of algorithms defining abnormal growth. Substantial empirical evidence shows that growth monitoring of children is suboptimal worldwide, with long diagnostic delays for target conditions [2–4], large numbers of futile referrals of children with normal variants of growth [5–7], and great variability in the definitions of abnormal growth and in practices [8,9]. Improving the performance of growth monitoring requires the standardization of definitions and practices as well as answers to two interlinked questions: What conditions should be targeted in priority? How do we define abnormal growth? The selection of the priority target conditions must precede the definition of abnormal growth by algorithms, because, as we have shown, their performance depends on the target conditions [10,11].

Six conditions have been included in the construction or validation of the algorithms currently proposed to define abnormal growth: growth hormone deficiency, celiac disease, cystic fibrosis, Turner syndrome, small-for-gestational-age (SGA) with no catch-up after two or three years, and renal tubular acidosis [10]. This very small number should be compared with the much longer lists (up to 118) of conditions that might affect growth proposed by medical professional groups [12–15]. This difference is explained by the dual nature of growth monitoring, that is, its two separate components: although the component associated with clinical expertise must take into account all of the conditions that might affect growth, that implemented in the stages of algorithm construction and validation can include the auxological data for only a limited number of conditions. Although the choice of these conditions has a strong effect on the performance of the algorithms, none of those proposed have been validated with auxological data of children affected by a large panel of conditions. This lack of validation limits their level of evidence, their implementation, and the standardization of practices. Moreover, because teams or societies of pediatric endocrinology have done most of the work on defining abnormal growth, endocrine conditions are overrepresented in these studies, at the expense of serious gastrointestinal and renal conditions. Crohn disease is an example of a gastrointestinal condition that raises serious problems of early identification [16].

The selection of priority target conditions for growth monitoring by algorithms must be based on rigorous criteria, such as those suggested by Wilson and Jungner to assess the relevance of screening programs [17]. The ideal typology of priority target condition has recently been the object of a systematic review by an international group of experts who set forth a four-point definition (see below). Our objective was to apply a formal consensus method to obtain a limited list of priority target conditions for growth monitoring by algorithms fulfilling these four criteria.

## Methods

### General methodology

**Fig 1:**
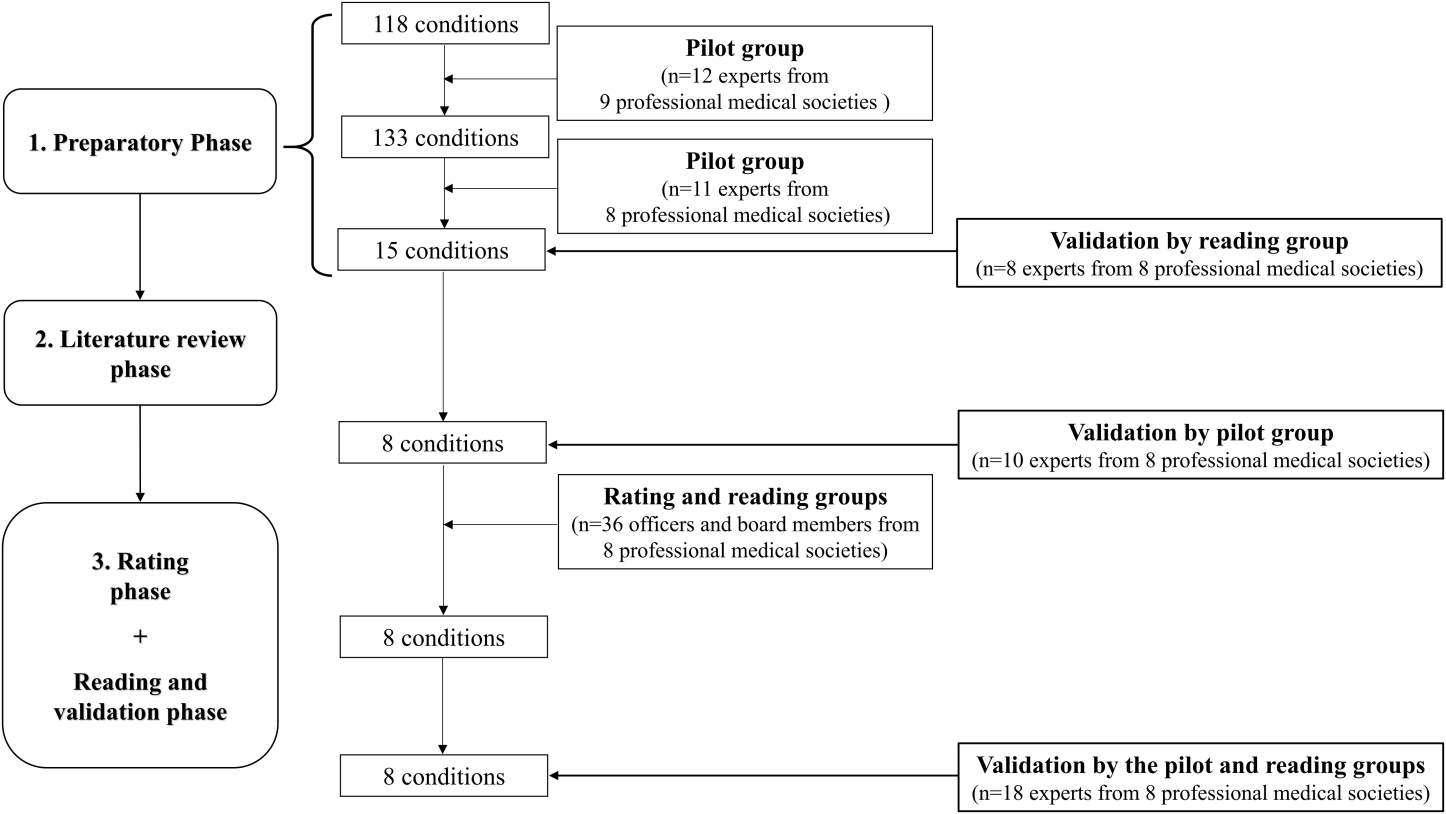
Different phases of the formal consensus process (modified version of the RAND/UCLA method).

We applied a formal consensus method using a modified version of the RAND/UCLA [18] method (Fig 1), which enabled us to combine the scientific data available in the literature and the experience of experts, and thus appeared to be an appropriate approach in view of the data available and the transversal nature of the expertise necessary. The modifications involved adding a preparatory phase, simplifying the rating scale and the requirements to reach a consensus, and changing the format of discussions. The consensus process was divided into three phases and used three separate working groups. The pilot group included 12 experts named by nine French professional medical societies generally considered to be concerned by the priority target conditions, both hospital subspecialists (in pediatric endocrinology, pediatric gastroenterology, and pediatric nephrology) and primary and secondary care physicians (general practitioners, public health pediatricians at maternal and child protection programs, school physicians, and private-practice and pediatric hospitalists) (S1 Table). The presidents of these nine professional medical societies served as the reading group (S1 Table). Officers and board members of these societies (n = 60) were asked to participate in the rating group (S1 Table). Representatives of three associations of parents of children with the priority target conditions were kept informed throughout the entire consensus process and could intervene at any point. No approval of an ethics committee/IRB was sought for this consensus process that did not involved human subject as participants.

### Ideal typology of priority target conditions

We had earlier obtained a national and then an international consensus of the definition of an ideal typology of priority target condition [10,19]. This definition was based on the criteria of Wilson and Jungner [17], adapted by Hall *et al*. in 2000 to the situation of growth monitoring in the form of a 16-item list [14] and subsequently modified and validated by us. A condition is considered to have an ideal typology for a priority target for growth monitoring by algorithms if: i) it is a health burden, that is, that the combination of its incidence and its severity is responsible for substantial morbidity and/or mortality; ii) it has a natural history including a long paucisymptomatic phase during which clinical expression is mainly auxological; iii) a relation between its early diagnosis and a more favorable outcome is well established; and iv) it has robust diagnostic criteria that are independent of the auxological parameters used to define abnormal growth. All four criteria were considered necessary.

### Consensus process

In the preparatory phase, the pilot group experts were asked to add potential target conditions for growth monitoring that they thought were missing from the diagnostic list of the *European Society for Paediatric Endocrinology* (ESPE), which classifies 118 conditions. Then, from this expanded list and on the basis of his or her personal expertise, each expert in the pilot group classified the 15 (arbitrarily selected number) conditions that he or she considered the highest priority target conditions for growth monitoring. This expert classification was synthesized by a simple weighting system. The reading group then reviewed this list of the 15 conditions with the highest scores to validate it.

The literature review phase allowed us to evaluate the evidence for each of these 15 conditions according to the four predefined criteria. This stage resulted in a new list, shorter and more specific, of conditions for which a sufficient level of evidence exist for each of these four criteria. The experts in the pilot group reviewed this evidence and evaluated this limited list.

During the rating phase, we submitted this new list to the rating group then to the reading group, by an online survey. These experts were asked to indicate for each target condition their degree of agreement (in 5 categories: disagree strongly, disagree somewhat, I am not an expert in this condition, agree somewhat, agree strongly) with the following statement: “this condition fulfills all four criteria described above for a priority target condition for growth monitoring”. The proportion of responses "agree somewhat" and "agree strongly" was calculated for each condition, with as a denominator the number of opinions expressed (excluding those stating they lacked the expertise). We considered *a priori* and arbitrarily that a strong consensus existed when this proportion exceeded 80%, a moderate consensus when it ranged from below 80 to 50%, and no consensus when the proportion was less than 50%. Several rounds of ratings, with feedback between them, were planned if necessary to reach a consensus. The consensus list was then validated by the pilot then reading groups.

## Results

### Preparatory phase

The 12 experts in the pilot group added 15 conditions to the ESPE list of 118. From this expanded list (n = 133), 11 of the 12 experts in the pilot group submitted an independent classification of the 15 conditions that seemed to them to be the highest priority targets for growth monitoring. We thus obtained the following list, in decreasing order of priority: celiac disease, Crohn disease, craniopharyngioma, chronic kidney disease, Turner syndrome, growth hormone deficiency, insufficient nutrient intake, psychosocial dwarfism, cystic fibrosis, hypercorticism, tubular disorders, cow’s milk protein allergy, cardiac disorders, hypothyroidism, and "cancer". This list was validated unanimously by the reading group (eight respondents among nine experts).

### Literature review phase

The analysis of the review literature led to the conclusion that 7 of these 15 conditions failed to meet at least one of the four criteria defining an ideal typology of priority target condition: cystic fibrosis, hypothyroidism, cardiac disorders, cow’s milk protein allergy, hypercorticism, insufficient nutrient intake, and psychosocial dwarfism. The criteria that these conditions failed to meet were as follows: i) low prevalence of undiagnosed cases, due to prenatal or neonatal screening (cystic fibrosis [20], congenital hypothyroidism [21], and cardiac disorders [22]) or the absence of a long paucisymptomatic phase during which the clinical expression is mainly auxological (acquired hypothyroidism [23], hypercorticism [24], cardiac disorders [25], and cow’s milk protein allergy [26]), and ii) the non-independence of the diagnostic criteria and the auxological parameters (insufficient nutrient intake [27], cow’s milk protein allergy [26] and psychosocial dwarfism [28]).

Moreover we added details for four of the target conditions. For the most frequent forms of growth hormone deficiency (those without certainty markers), the diagnostic criterion is not independent of the auxological parameters used to define abnormal growth since it very frequently involves height velocity. We therefore decided to specify "growth hormone deficiency associated with a pituitary stalk interruption syndrome", which requires diagnostic confirmation by cerebral imaging [29]. Among the numerous causes of chronic kidney disease and tubular disorders in children, some are the object of prenatal ultrasound screening (e.g., obstructive uropathy [30]); others do not have a long paucisymptomatic phase during which the clinical expression is mainly auxological (e.g., Bartter syndrome). We thus decided to restrict the chronic kidney diseases to juvenile nephronophthisis and the tubular disorders to infantile cystinosis. Finally, although all cancers can result in restriction of weight or height growth, the duration of the paucisymptomatic phase varies substantially according to the type of cancer [31]. We decided to limit cancer types to hypothalamic-optochiasmatic astrocytoma, which include Russell syndrome (i.e., diencephalic syndrome).

We thus retained eight target conditions fulfilling the four criteria defining an ideal typology of priority target condition: celiac disease, Crohn disease, craniopharyngioma, juvenile nephronophthisis, Turner syndrome, growth hormone deficiency with pituitary stalk interruption syndrome, infantile cystinosis, and hypothalamic-optochiasmatic astrocytoma. S2–S5 Tables in the appendix report the literature supporting the existence of these criteria for these eight conditions. This limited list of target conditions was validated unanimously, after discussion, by the experts of the pilot group.

### Rating phase

Of the 68 experts from the rating group (n = 60) and the reading group (n = 8) who were asked to participate in this online survey, 36 (53%) did so, including 28 members (47%) of the rating group and 8 (100%) of the reading group (Table 1 and S1 Table). The eight conditions were assessed as priority target conditions by a strong expert consensus on the first round of the survey (Table 1 and S1 Table).

**Table 1:** Results of the rating phase of the target conditions judged to be priorities for growth monitoring (n = 36 experts of 68).

Target conditions	Number of responses to the question: “This condition fulfills all four criteria described above for a priority target condition for growth monitoring”					Total number of opinions expressed^†^	% of favorable opinions^‡^	Level of consensus obtained
	Disagree strongly	Disagree somewhat	I don't have expertise	Agree somewhat	Agree strongly			
Celiac disease	0	3	2	7	24	34	91	Strong
Crohn's disease	0	3	3	8	22	33	91	Strong
Craniopharyngioma	0	1	6	11	18	30	97	Strong
Juvenile nephronophthisis	0	1	23	8	4	13	92	Strong
Turner syndrome	0	0	3	9	24	33	100	Strong
Growth hormone deficiency with pituitary stalk interruption syndrome	0	0	5	4	27	31	100	Strong
Infantile cystinosis	0	2	22	7	5	14	86	Strong
Hypothalamic-optochiasmatic astrocytoma	0	2	14	12	8	22	91	Strong

^†^ excluding the opinions reporting a lack of expertise;

^‡^ the proportion of responses "agree somewhat" and "agree strongly" was calculated for each condition, with all of the opinions expressed (except lack of expertise) as the denominator.

## Discussion

We report the results of the first formal consensus process to obtain a limited list of priority target conditions for algorithms for monitoring children's growth. The expertise of a panel of professionals representing the entire set of participants in monitoring children's growth together with a review of the literature enabled us to identify eight conditions meeting the four predefined criteria defining an ideal typology of a priority target condition. The resulting list of priority target conditions was validated with a strong degree of agreement by a panel of experts. Of these eight conditions, four (growth hormone deficiency with pituitary stalk interruption syndrome, Turner syndrome, celiac disease, and infantile cystinosis) have previously been included in the construction or validation of the seven algorithms proposed in the literature to define abnormal growth, and six (growth hormone deficiency with pituitary stalk interruption syndrome, Turner syndrome, juvenile nephronophthisis, celiac disease, Crohn disease, and infantile cystinosis) have already been included on the lists of target conditions proposed by the authors of the Dutch consensus and the Coventry consensus [12,14]. Craniopharyngioma and hypothalamic-optochiasmatic astrocytoma have never before been included on these lists, despite data indicating that diagnostic delays can last up to several years and that auxological signs precede neuro-ophthalmologic signs [4].

The consensus process did not include several conditions frequently included in the construction or validation of algorithms (cystic fibrosis and SGA with no catch-up after two or three years) or in lists of target conditions or pediatrics textbooks (insufficient nutrient intake, psychosocial dwarfism, cow’s milk protein allergy, hypothyroidism, and hypercorticism). The reasons these conditions were not selected are related to the low prevalence of undiagnosed cases in France because of neonatal screening, as for cystic fibrosis, to the lack of independence of the diagnostic criteria compared with auxological parameters, as for insufficient nutrient intake, and to the absence of a long paucisymptomatic phase during which the clinical expression is mainly auxological, as for hypercorticism. It is important here to recall that the objective of this consensus is not to replace clinical expertise but to select conditions that should be included in the construction of algorithms for defining abnormal growth, which will be used as a complement and not a replacement for this clinical expertise. Clinicians must of course keep these conditions in mind during their semiotic analysis of the child's growth and health, even though they have not been included in this consensus list, so that they can be identified early.

The consensus process that we report has some limitations. First, although the working groups included both primary and secondary care physicians and hospital subspecialists (pediatric endocrinologists, pediatric gastroenterologists, and pediatric nephrologists), we did not include all pediatric subspecialties (e.g., pediatric oncology), an omission that might have modified the results of the consensus process. This restriction did not prevent the selection of target conditions related to non-represented subspecialties (e.g., hypothalamic-optochiasmatic astrocytoma). The role of primary and secondary care physicians in the consensus process could be questionable given their partial expertise to select priority target conditions for growth monitoring. However, international recommendations for the development of practice guidelines emphasize the need for participation of all relevant professional groups [32], and primary and secondary care physicians have a pivotal role in growth monitoring. Second, we modified the RAND/UCLA method by adding a preparatory phase to reduce the ESPE 118-item list to 15 target conditions before the rating phase, simplifying the rating scale and the requirements to reach a consensus, and by changing the format of the discussions, which were electronic rather than face-to-face for reasons of feasibility. Third, the attrition of experts during the rating phase was substantial (47%) but also usual for this type of consensus. It may in part be explained by the difficulty of highly subspecialized physicians in expressing an opinion about conditions outside their own specialty. Fourth, this consensus might have been affected by the "opinion leader" effect during the literature review phase within the pilot group, with a smaller number of participants (n = 11). Fifth, representatives of parent associations had limited contribution to the medical and scientific process. However, they were present during all discussions and their implication in the process will allow for considering their views and preferences for future screening tools developed from the obtained consensus, as recommended [32].

A consensus about the priority target conditions for growth monitoring by algorithms must be able to be adapted to screening activities already existing in each country. Here, we took into account the shifts resulting from the introduction of routine neonatal screening for cystic fibrosis in France. In countries where this screening is not routine, as in the Netherlands, cystic fibrosis would very probably be considered a priority target condition for growth monitoring by algorithms. Similarly, the potential dissemination of antenatal screening techniques for trisomy 21 by karyotyping fetal blood cells in the circulating peripheral blood is likely to result in a collapse in the number of undiagnosed cases of Turner syndrome, which may raise questions in the years to come about the inclusion of this condition on this list of priority target conditions.

In conclusion, our work has produced a first formalized consensus of eight priority target conditions for growth monitoring by algorithms, validated nationally by a multidisciplinary working group. The method for obtaining this consensus could be re-used to build an interdisciplinary consensus on the international scale. This consensus will also allow for standardization of the construction and validation process of algorithms defining abnormal growth.

## Supporting information

S1 Table Repartition of experts from pilot, rating, and reading groups by according to the nine French professional medical societies involved in the consensus process. (DOC)

S2 Table Evidence supporting the health burden of conditions selected as priority targets for children’s growth monitoring by algorithms. (DOC)

S3 Table Evidence supporting the existence of a long paucisymptomatic phase during which the clinical expression is mainly auxological for conditions selected as priority targets for children’s growth monitoring by algorithms. (DOC)

S4 Table Evidence supporting the existence of a relation between early diagnosis and prognosis for conditions selected as priority targets for children’s growth monitoring by algorithms. (DOC)

S5 Table Evidence supporting the presence of diagnostic criteria independent of auxological parameters for conditions selected as priority targets for children’s growth monitoring by algorithms. (DOC)
