# Supplementary material for: Priority target conditions for algorithms for monitoring children's growth: Interdisciplinary consensus
Source: PLoS One. 2017 Apr 27;12(4):e0176464. doi: 10.1371/journal.pone.0176464 (PMC5407643; doi:10.1371/journal.pone.0176464)
Supplement: S1 Table — (DOC) [file pone.0176464.s001.doc]

**S1 Table.** Repartition of experts from pilot, rating, and reading groups by according to the nine French professional medical societies involved in the consensus process.

|  | Pilot group | Reading group | Rating group |
| --- | --- | --- | --- |
| **French Society for Pediatric Endocrinology and Diabetology** | 2 | 1 | 5/6 |
| **French Society for Pediatric Nephrology** | 1 | 1 | 2/4 |
| **French Group for Pediatric Hepatology, Gastroenterology, and Nutrition** | 2 | 1 | 3/8 |
| **French Association for Primary Care Pediatrics** | 1 | 1 | 9/14 |
| **French Society of General Medicine** | 2 | 1 | 0/9 |
| **Society for Therapeutic Training of General Practitioners** | 1 | 1 | 3/8 |
| **General pediatrics group of the French Society of Pediatrics** | 1 | 1 | 5/9 |
| **Committee on Public Health and Social Pediatrics of the French Society of Pediatrics** | 1 | 1 | 1/2 |
| **French Association for Health Promotion in Schools and Universities** | 1 | - | - |
| **Total** | **12** | **8/9** | **36/60** |
