# Supplementary material for: Priority target conditions for algorithms for monitoring children's growth: Interdisciplinary consensus
Source: PLoS One. 2017 Apr 27;12(4):e0176464. doi: 10.1371/journal.pone.0176464 (PMC5407643; doi:10.1371/journal.pone.0176464)
Supplement: S2 Table — (DOC) [file pone.0176464.s002.doc]

**S2 Table. Evidence supporting the health burden of conditions selected as priority targets for children’s growth monitoring by algorithms.**

| **Conditions** | **Incidence** | **Prevalence** | **Mortality** | **Potentially avoidable morbidity with early treatment** |
| --- | --- | --- | --- | --- |
| **Celiac disease** | [10:100 000 – 17.4:100 000] | [1:270 – 1:1000] | SMR = [1.37; 3.4] | Malabsorption, increase risk of cancer and autoimmune diseases |
| **Crohn disease** | [5:100 000 – 9.4:100 000] | [40:100 000 – 124:100 000] | SMR = [1.10; 1.40] | Digestive abscesses, fistula, or infections, short stature, malnutrition, increase of cancer risks. |
| **Craniopharyngioma** | [0.13:100 000 – 2:100 000] | [1:100 000 – 3:100 000] | SMR = [8.75; 17] | Visual, endocrine disorders, or neurological sequelae |
| **Turner syndrome** | 40:100 000 | 50:100 000 | SMR = [2.86; 3] | Short stature |
| **Growth hormone deficiency   with PSIS** | [1:4000 – 1:10 000] | [14.7:100 000 – 27:100 000] | SMR = 3.8 | Short stature, hypoglycemia, metabolic disorders, or cortisol deficiency |
| **Infantile cystinosis** | [0.5:100 000 – 1:100 000] | 0.5:100 000 | Mortality rate: 28% | Chronic kidney disease, endocrine disorders, visual disorders, or metabolic disorders |
| **Juvenile nephronophthisis** | [0.10:100 000 – 2:100 000] | - | - | Chronic kidney disease |
| **Hypothalamic-optochiasmatic   astrocytoma** | - | 1:100 000 | Mortality rate: 44% | Visual, endocrine, cognitive or neurological sequelae |
| [range]. PSIS: pituitary stalk interruption syndrome; SMR: standardized mortality ratio. | | | | |

# **REFERENCES OF APPENDICES**

1. Holtmeier W, Caspary WF. Celiac disease. Orphanet J Rare Dis. 2006;1: 3.

2. Dydensborg S, Toftedal P, Biaggi M, Lillevang ST, Hansen DG, Husby S. Increasing prevalence of coeliac disease in Denmark: a linkage study combining national registries. Acta Paediatr. 2012;101: 179-184.

3. Ludvigsson JF, Rubio-Tapia A, van Dyke CT, Melton LJ, Zinsmeister AR, Lahr BD, et al. Increasing incidence of celiac disease in a North American population. Am J Gastroenterol. 2013;108: 818-824.

4. Ludvigsson JF. Mortality and malignancy in celiac disease. Gastrointest Endosc Clin N Am. 2012;22: 705-722.

5. Corrao G, Corazza GR, Bagnardi V, Brusco G, Ciacci C, Cottone M, et al. Mortality in patients with coeliac disease and their relatives: a cohort study. Lancet. 2001;358: 356-361.

6. Goddard CJ, Gillett HR. Complications of coeliac disease: are all patients at risk? Postgrad Med J. 2006;82: 705-712.

7. Manninen P, Karvonen AL, Huhtala H, Rasmussen M, Collin P. The epidemiology of inflammatory bowel diseases in Finland. Scand J Gastroenterol. 2010;45: 1063-1067.

8. Manninen P, Karvonen AL, Huhtala H, Rasmussen M, Salo M, Mustaniemi L, et al. Mortality in ulcerative colitis and Crohn's disease. A population-based study in Finland. J Crohns Colitis. 2012;6: 524-528.

9. Jussila A, Virta LJ, Pukkala E, Farkkila MA. Mortality and causes of death in patients with inflammatory bowel disease: a nationwide register study in Finland. J Crohns Colitis. 2014;8: 1088-1096.

10. Duricova D, Pedersen N, Elkjaer M, Gamborg M, Munkholm P, Jess T. Overall and cause-specific mortality in Crohn's disease: a meta-analysis of population-based studies. Inflamm Bowel Dis. 2010;16: 347-353.

11. Peneau A, Savoye G, Turck D, Dauchet L, Fumery M, Salleron J, et al. Mortality and cancer in pediatric-onset inflammatory bowel disease: a population-based study. Am J Gastroenterol. 2013;108: 1647-1653.

12. Garnett MR, Puget S, Grill J, Sainte-Rose C. Craniopharyngioma. Orphanet J Rare Dis. 2007;2: 18.

13. Bunin GR, Surawicz TS, Witman PA, Preston-Martin S, Davis F, Bruner JM. The descriptive epidemiology of craniopharyngioma. J Neurosurg. 1998;89: 547-551.

14. Crowley RK, Hamnvik OP, O'Sullivan EP, Behan LA, Smith D, Agha A, et al. Morbidity and mortality in patients with craniopharyngioma after surgery. Clin Endocrinol (Oxf). 2010;73: 516-521.

15. Olsson DS, Andersson E, Bryngelsson IL, Nilsson AG, Johannsson G. Excess mortality and morbidity in patients with craniopharyngioma, especially in patients with childhood onset: a population-based study in Sweden. J Clin Endocrinol Metab. 2015;100: 467-474.

16. Visser J, Hukin J, Sargent M, Steinbok P, Goddard K, Fryer C. Late mortality in pediatric patients with craniopharyngioma. J Neurooncol. 2010;100: 105-111.

17. Gravholt CH, Juul S, Naeraa RW, Hansen J. Morbidity in Turner syndrome. J Clin Epidemiol. 1998;51: 147-158.

18. Stochholm K, Juul S, Juel K, Naeraa RW, Gravholt CH. Prevalence, incidence, diagnostic delay, and mortality in Turner syndrome. J Clin Endocrinol Metab. 2006;91: 3897-3902.

19. Schoemaker MJ, Swerdlow AJ, Higgins CD, Wright AF, Jacobs PA. Mortality in women with Turner syndrome in Great Britain: a national cohort study. J Clin Endocrinol Metab. 2008;93: 4735-4742.

20. Vimpani GV, Vimpani AF, Lidgard GP, Cameron EH, Farquhar JW. Prevalence of severe growth hormone deficiency. Br Med J. 1977;2: 427-430.

21. Mills JL, Schonberger LB, Wysowski DK, Brown P, Durako SJ, Cox C, et al. Long-term mortality in the United States cohort of pituitary-derived growth hormone recipients. J Pediatr. 2004;144: 430-436.

22. Wang Q, Hu Y, Li G, Sun X. Pituitary stalk interruption syndrome in 59 children: the value of MRI in assessment of pituitary functions. Eur J Pediatr. 2014;173: 589-595.

23. Gascoin-Lachambre G, Brauner R, Duche L, Chalumeau M. Pituitary stalk interruption syndrome: diagnostic delay and sensitivity of the auxological criteria of the growth hormone research society. PLoS One. 2011;6: e16367.

24. Wuhl E, van Stralen KJ, Wanner C, Ariceta G, Heaf JG, Bjerre AK, et al. Renal replacement therapy for rare diseases affecting the kidney: an analysis of the ERA-EDTA Registry. Nephrol Dial Transplant. 2014;29 Suppl 4: iv1-8.

25. Viltz L, Trauner DA. Effect of age at treatment on cognitive performance in patients with cystinosis. J Pediatr. 2013;163: 489-492.

26. Elmonem MA, Veys K, Soliman N, Van Dyck M, Van Den Heuvel L, Levtchenko E. Cystinosis : a review. Orphanet Journal of Rare Diseases 2016.

27. Emma F, Nesterova G, Langman C, Labbe A, Cherqui S, Goodyer P, et al. Nephropathic cystinosis: an international consensus document. Nephrol Dial Transplant. 2014;29 Suppl 4: iv87-94.

28. Brodin-Sartorius A, Tete MJ, Niaudet P, Antignac C, Guest G, Ottolenghi C, et al. Cysteamine therapy delays the progression of nephropathic cystinosis in late adolescents and adults. Kidney Int. 2012;81: 179-189.

29. Simms RJ, Eley L, Sayer JA. Nephronophthisis. Eur J Hum Genet. 2009;17: 406-416.

30. Ala-Mello S, Koskimies O, Rapola J, Kaariainen H. Nephronophthisis in Finland: epidemiology and comparison of genetically classified subgroups. Eur J Hum Genet. 1999;7: 205-211.

31. Singhal S, Birch JM, Kerr B, Lashford L, Evans DG. Neurofibromatosis type 1 and sporadic optic gliomas. Arch Dis Child. 2002;87: 65-70.

32. British Neuro-Oncology Society (2011) Guidelines on the diagnosis and management of Optic Pathway Glioma (OPG)

33. Kilday JP, Bartels U, Huang A, Barron M, Shago M, Mistry M, et al. Favorable survival and metabolic outcome for children with diencephalic syndrome using a radiation-sparing approach. J Neurooncol. 2014;116: 195-204.
