# Supplementary material for: Priority target conditions for algorithms for monitoring children's growth: Interdisciplinary consensus
Source: PLoS One. 2017 Apr 27;12(4):e0176464. doi: 10.1371/journal.pone.0176464 (PMC5407643; doi:10.1371/journal.pone.0176464)
Supplement: S3 Table — (DOC) [file pone.0176464.s003.doc]

**S3 Table.** Evidence supporting the existence of a long paucisymptomatic phase during which the clinical expression is mainly auxological for conditions selected as priority targets for children’s growth monitoring by algorithms**.**

| **Conditions** | **Age at diagnosis** | **Auxological symptoms at diagnosis** | | **Auxological symptoms  before diagnosis** | | **Potential reductions in time to diagnosis^*^** | |  |
| --- | --- | --- | --- | --- | --- | --- | --- | --- |
| **Celiac disease** | **Median**:  6.2 years (0.9; 15.9) (girls)  7.1 years (0.8; 16.1) (boys) | Mean standardized height:  -0.45 (± 1.1) SD (girls)  -0.58 (± 1.2) SD (boys)  Mean standardized BMI:  -0.25 (± 1.2) SD (girls)  -0.44 (± 1.1) SD (boys)  Mean distance to standardized target height:  -0.57 (± 1.2) SD (girls)  -0.67 (± 1.1) SD (boys) | | Growth retardation of 2 years before diagnosis:  57% (girls)  48% (boys) | | **Median**:  3.2 years (0; 4.9) (girls)  2.7 years (0; 4.9) (boys) | |  |
| **Crohn disease** | **Median**:  12.9 years  [10.8; 14.3] | Mean standardized height:  -0.54 SD 95% CI [-0.67; -0.41]  Mean standardized weight:  -1.06 SD 95% CI [-1.21; -0.92]  Height retardation:  (< 10^th^ p): 17%  (< 3^th^ p): 13%  (< 0.04^th^ p): 2%  Weight retardation: (< 10^th^ p): 32%  (< 3^th^ p): 27%  (< 0.04^th^ p): 7% | | Height and weight retardation: (< 5^th^ p or < -2 SD or absolute deflection < 2 SD) : 16%  Weight retardation: 61% | | **Median**: 8 months [4; 16] | |  |
| **Craniopharyngioma** | **Median**:  [7; 8.3] years | Mean standardized height: -1 SD  Mean standardized BMI: +1 SD | | Height retardation and absolute height deflection from 10 to 12 months  Height retardation: 80% | | **Median**: 2 years (0.25; 9)  **Median** (height criterion):  1.5 years [1.5; 3.5]  **Median** (BMI criterion):  3.5 years [1.5; 4.5] | |  |
| (min: max); [Q1; Q3]. BMI: body mass index; 95% CI: 95% confidence interval; 0.04^th^ p: 0.04^th^ percentile; 3^th^ p: 3^th^ percentile; 5^th^ p: 5^th^ percentile; 10^th^ p: 10^th^ percentile; SD: standard deviation. ^*^ The theoretical reduction in time to diagnosis is the difference between the real age at diagnosis and the first main auxological symptoms. | | | | | | | |  |
| **Conditions** | **Age at diagnosis** | **Auxological symptoms at diagnosis** | | **Auxological symptoms  before diagnosis** | | **Potential reductions in time to diagnosis^*^** | |  |
| **Turner syndrome** | **Median**:  10 years | | Mean standardized height: -2.9 SD (± 1.4) | | Height retardation at 4 years (< 5^th^ p): 68.4% | | **Mean**: 5.2 years | |
| **Growth hormone deficiency   with PSIS** | **Median**:  [2.5; 4.8] years | | Median standardized height: -2.5 SD (-4.3; -1.3) [-3.5; -2]  Median standardized weight: -2.4 SD (-4; -1.1) [-2.8; -1]  Median standardized BMI: -0.23 SD (-3.7; 4) [-1.1; 0.5]  Median standardized height velocity:  -3.1 SD (-4.2; 0.3) [-3.4; -1.6] | | Height retardation (GHRS criteria): 100% | | **Median**: [2; 2.3] years | |
| **Infantile cystinosis** | **Median**:  [18; 21] months | | Standardized height: -2 SD  Standardized weight: -2 SD  Weight loss: -8% | | Height retardation (< 3^th^ p)  from 6 to 12 months  Height and weight retardation (first symptom): [63% - 82.6%] | | 15 months | |
| **Juvenile nephronophthisis** | 9.4 (± 3) years | | - | | Height and weight retardation (first symptom): 13% | | **Mean**: 1.5 years^**^ | |
| **Hypothalamic-  optochiasmatic astrocytoma** | 4.5 years [2.5; 7.5] | | - | | Height retardation: 66.6% | | **Median**(height criterion):  1.5 years [1; 1.5]  **Median**(BMI criterion):  2.5 years [2.5; 2.7] | |
| Russell syndrome | [0.54; 1.5] years | | Mean standardized weight: -2.8 SD (-0.73; -3.89)  Mean standardized BMI: -3.5 SD (± 0.2)  Weight retardation (< -2 SD): 81.8%  Weight retardation (< 5^th^ p): [75% - 77.7%] | | Mean age at onset of height and weight retardation: 14 months (1; 32) | | **Mean**: [12; 12.5] months | |
| Optic pathway glioma | 3.6 years [2.5; 7.7] | | - | | Height retardation: 50% | | **Median**(height criterion):  1.5 years [0.5; 2]  **Median**(BMI criterion):  1.5 years [0.5; 2.5] | |
| (min; max); [Q1; Q3]. BMI: body mass index; 5^th^ p: 5^th^ percentile; GHRS: Growth Hormone Research Society; PSIS: pituitary stalk interruption syndrome; SD: standard deviation.  ^*^ The theoretical reductions in time to diagnosis is the difference between the real age at diagnosis and the main first auxological symptoms;^**^ In this study, the theoretical reduction in time to diagnosis is the difference between the real age at diagnosis and the first main symptoms or signs such as anemia, height and weight retardation, polydipsia/polyuria, or hypertension. | | | | | | | |  |

# **REFERENCES OF APPENDICES**

1. Saari A, Harju S, Makitie O, Saha MT, Dunkel L, Sankilampi U. Systematic growth monitoring for the early detection of celiac disease in children. JAMA Pediatr. 2015;169: e1525.

2. Sawczenko A, Sandhu BK. Presenting features of inflammatory bowel disease in Great Britain and Ireland. Arch Dis Child. 2003;88: 995-1000.

3. Timmer A, Behrens R, Buderus S, Findeisen A, Hauer A, Keller KM, et al. Childhood onset inflammatory bowel disease: predictors of delayed diagnosis from the CEDATA German-language pediatric inflammatory bowel disease registry. J Pediatr. 2011;158: 467-473.

4. Taylor M, Couto-Silva AC, Adan L, Trivin C, Sainte-Rose C, Zerah M, et al. Hypothalamic-pituitary lesions in pediatric patients: endocrine symptoms often precede neuro-ophthalmic presenting symptoms. J Pediatr. 2012;161: 855-863.

5. Muller HL, Emser A, Faldum A, Bruhnken G, Etavard-Gorris N, Gebhardt U, et al. Longitudinal study on growth and body mass index before and after diagnosis of childhood craniopharyngioma. J Clin Endocrinol Metab. 2004;89: 3298-3305.

6. Hoffmann A, Boekhoff S, Gebhardt U, Sterkenburg AS, Daubenbuchel AM, Eveslage M, et al. History before diagnosis in childhood craniopharyngioma: associations with initial presentation and long-term prognosis. Eur J Endocrinol. 2015;173: 853-862.

7. Savendahl L, Davenport ML. Delayed diagnoses of Turner's syndrome: proposed guidelines for change. J Pediatr. 2000;137: 455-459.

8. Gascoin-Lachambre G, Brauner R, Duche L, Chalumeau M. Pituitary stalk interruption syndrome: diagnostic delay and sensitivity of the auxological criteria of the growth hormone research society. PLoS One. 2011;6: e16367.

9. Bar C, Zadro C, Diene G, Oliver I, Pienkowski C, Jouret B, et al. Pituitary stalk interruption syndrome from infancy to adulthood: clinical, hormonal, and radiological assessment according to the initial presentation. PLoS One. 2015;10: e0142354.

10. Pinto G, Adan L, Souberbielle JC, Thalassinos C, Brunelle F, Brauner R. Idiopathic growth hormone deficiency: presentation, diagnostic and treatment during childhood. Ann Endocrinol (Paris). 1999;60: 224-231.

11. Brodin-Sartorius A, Tete MJ, Niaudet P, Antignac C, Guest G, Ottolenghi C, et al. Cysteamine therapy delays the progression of nephropathic cystinosis in late adolescents and adults. Kidney Int. 2012;81: 179-189.

12. Bertholet-Thomas A, Llanas B, Servais A, Bendelac N, Goizet C, Choukroun G, et al. [Significance of the urine strip test in case of stunted growth]. Arch Pediatr. 2015;22: 756-762.

13. Greco M, Brugnara M, Zaffanello M, Taranta A, Pastore A, Emma F. Long-term outcome of nephropathic cystinosis: a 20-year single-center experience. Pediatr Nephrol. 2010;25: 2459-2467.

14. Elmonem MA, Veys K, Soliman N, Van Dyck M, Van Den Heuvel L, Levtchenko E. Cystinosis : a review. Orphanet Journal of Rare Diseases 2016.

15. Nesterova G, Gahl W. Nephropathic cystinosis: late complications of a multisystemic disease. Pediatr Nephrol. 2008;23: 863-878.

16. Ala-Mello S, Koskimies O, Rapola J, Kaariainen H. Nephronophthisis in Finland: epidemiology and comparison of genetically classified subgroups. Eur J Hum Genet. 1999;7: 205-211.

17. Fleischman A, Brue C, Poussaint TY, Kieran M, Pomeroy SL, Goumnerova L, et al. Diencephalic syndrome: a cause of failure to thrive and a model of partial growth hormone resistance. Pediatrics. 2005;115: e742-748.

18. Brauner R, Trivin C, Zerah M, Souberbielle JC, Doz F, Kalifa C, et al. Diencephalic syndrome due to hypothalamic tumor: a model of the relationship between weight and puberty onset. J Clin Endocrinol Metab. 2006;91: 2467-2473.

19. Kilday JP, Bartels U, Huang A, Barron M, Shago M, Mistry M, et al. Favorable survival and metabolic outcome for children with diencephalic syndrome using a radiation-sparing approach. J Neurooncol. 2014;116: 195-204.

20. Poussaint TY, Barnes PD, Nichols K, Anthony DC, Cohen L, Tarbell NJ, et al. Diencephalic syndrome: clinical features and imaging findings. AJNR Am J Neuroradiol. 1997;18: 1499-1505.

21. Sardi I, Bresci C, Schiavello E, Biassoni V, Fratoni V, Cardellicchio S, et al. Successful treatment with a low-dose cisplatin--etoposide regimen for patients with diencephalic syndrome. J Neurooncol. 2012;109: 375-383.
