# Supplementary material for: Priority target conditions for algorithms for monitoring children's growth: Interdisciplinary consensus
Source: PLoS One. 2017 Apr 27;12(4):e0176464. doi: 10.1371/journal.pone.0176464 (PMC5407643; doi:10.1371/journal.pone.0176464)
Supplement: S4 Table — (DOC) [file pone.0176464.s004.doc]

**S4 Table.** Evidence supporting the existence of a relation between early diagnosis and prognosis for conditions selected as priority targets for children’s growth monitoring by algorithms**.**

| **Conditions** | **Treatments** | **Prognostic judgment criteria** | **Relation between early diagnosis and prognosis** |
| --- | --- | --- | --- |
| **Celiac disease** | Gluten-free diet | Mortality | Diagnostic delays ≥ 10 years (25%):  SMR = 3.8; 95% CI [2.2; 6.4] |
|  |  | BMI | Early diagnosis is associated with a favorable change in BMI OR = 2.1; 95% CI [1.2; 3.6] |
|  |  | Autoimmune disorders | Early diagnosis (< 2 years vs. > 20 years) is associated with a low prevalence of autoimmune disorders (5.1% vs. 34.0%) |
| **Crohn disease** | Various | Adult height | Long diagnostic delay is associated with short height at diagnosis (r = -0.22; p = 0.032)  Height at diagnosis is associated with adult height (r = 0.75; p < 0.01) |
| **Craniopharyngioma** | Surgery/radiotherapy | Morbidity | Long diagnostic delay (9 months) is associated with endocrine disorders at diagnosis (p = 0.01) |
| **Turner syndrome** | Growth hormone | Height gain | Height gain (treated vs. control): + 7.2 cm 95% CI [6.0; 8.4]  Height gain is associated with age at start of treatment  (β = -0.33)  Early treatment is associated with height gain  (r² = 0.29; p < 0.01) |
|  |  | Adult height | Age at start of treatment is associated with adult height (β = -3.06; 95% CI [-3.49; -2.63])  Duration of treatment is associated with adult height  (β = 1.54; 95% CI [1.22; 1.85]) |
| BMI: body mass index; 95% CI: 95% confidence interval; OR: odds ratio; SD: standard deviation; SMR: standardized mortality ratio. | | | |

| **Conditions** | **Treatments** | **Prognostic judgment criteria** | | **Relation between early diagnosis and prognosis** |
| --- | --- | --- | --- | --- |
| **Growth hormone deficiency   with PSIS** | Growth hormone | Height gain | Early treatment (< 4 vs. > 4 years) is associated with height gain during the first 1 year of treatment and overall height gain (p < 0.01)  Height at start of treatment is negatively associated with height gain during the first 2 years of treatment (p < 0.01)  Age at start of treatment is negatively associated with overall height gain (r = -0.37; p < 0.01)  Prepubertal height gain is associated with overall height gain  (r = 0.75; p < 0.01) | |
|  |  | Adult height | Age is negatively associated with growth velocity during treatment  (r = -0.38; p < 0.01) | |
| **Infantile cystinosis** | Cysteamine | Renal function/GFR | Late treatment is associated with strong risk of developing stage III chronic kidney disease (HR = 1.32; 95% CI [1.09; 1.61])  Early treatment (< 2 years vs. > 2 years or not treated) is associated with better GFR  Early treatment (< 2 years vs. > 2 years or not treated) is associated with age of late-onset end-stage kidney disease (74 years vs. 10 years) | |
|  |  | Morbidity/mortality | Early treatment (< 5 years vs. > 5 years or not treated) is associated with a low prevalence of diabetes, hypothyroidism, and neuromuscular disorders (p < 0.01)  Early treatment (< 5 years vs. > 5 years or not treated) is associated with a lower mortality rate (p < 0.01) | |
|  |  | Cognitive performances | Early treatment (< 2 years vs. > 2 years) is associated with better cognitive performance (p < 0.05) | |
| 95% CI: 95% confidence interval; GFR: glomerular filtration rate; HR: hazard ratio; PSIS: pituitary stalk interruption syndrome. | | | | |

| **Conditions** | **Treatments** | **Prognostic judgment criteria** | **Relation between early diagnosis and prognosis** |
| --- | --- | --- | --- |
| **Juvenile nephronophthisis** | Blood-pressure control | Renal function/GFR | Progression of chronic kidney disease at 5 years is associated with blood-pressure control (intensified vs. conventional):  HR = 0.65; 95% CI [0.44; 0.94]  Age at start of treatment is negatively associated with progression of chronic kidney disease (increase of DFG) (r²=0.02) |
|  | Low-protein diet | Renal function/GFR | Age at start of treatment is negatively associated with progression of chronic kidney disease at 2 years  (r²=0.01; p=0.01) |
| **Hypothalamic-optochiasmatic   astrocytoma** | Surgery/radiotherapy/chemotherapy | Progression-free survival/recurrence or Overall survival | No data |
| 95% CI: 95% confidence interval; HR: hazard ratio; GFR: glomerular filtration rate; HR: hazard ratio. | | | |

# **REFERENCES OF APPENDICES**

1. Corrao G, Corazza GR, Bagnardi V, Brusco G, Ciacci C, Cottone M, et al. Mortality in patients with coeliac disease and their relatives: a cohort study. Lancet. 2001;358: 356-361.

2. Ukkola A, Maki M, Kurppa K, Collin P, Huhtala H, Kekkonen L, et al. Changes in body mass index on a gluten-free diet in coeliac disease: a nationwide study. Eur J Intern Med. 2012;23: 384-388.

3. Ventura A, Magazzu G, Greco L. Duration of exposure to gluten and risk for autoimmune disorders in patients with celiac disease. SIGEP Study Group for Autoimmune Disorders in Celiac Disease. Gastroenterology. 1999;117: 297-303.

4. Sawczenko A, Ballinger AB, Savage MO, Sanderson IR. Clinical features affecting final adult height in patients with pediatric-onset Crohn's disease. Pediatrics. 2006;118: 124-129.

5. Hoffmann A, Boekhoff S, Gebhardt U, Sterkenburg AS, Daubenbuchel AM, Eveslage M, et al. History before diagnosis in childhood craniopharyngioma: associations with initial presentation and long-term prognosis. Eur J Endocrinol. 2015;173: 853-862.

6. Stephure DK. Impact of growth hormone supplementation on adult height in Turner syndrome: results of the Canadian randomized controlled trial. J Clin Endocrinol Metab. 2005;90: 3360-3366.

7. Ranke MB, Lindberg A, Ferrandez Longas A, Darendeliler F, Albertsson-Wikland K, Dunger D, et al. Major determinants of height development in Turner syndrome (TS) patients treated with GH: analysis of 987 patients from KIGS. Pediatr Res. 2007;61: 105-110.

8. Quigley CA, Crowe BJ, Anglin DG, Chipman JJ. Growth hormone and low dose estrogen in Turner syndrome: results of a United States multi-center trial to near-final height. J Clin Endocrinol Metab. 2002;87: 2033-2041.

9. Soriano-Guillen L, Coste J, Ecosse E, Leger J, Tauber M, Cabrol S, et al. Adult height and pubertal growth in Turner syndrome after treatment with recombinant growth hormone. J Clin Endocrinol Metab. 2005;90: 5197-5204.

10. Tauber M, Chevrel J, Diene G, Moulin P, Jouret B, Oliver I, et al. Long-term evolution of endocrine disorders and effect of GH therapy in 35 patients with pituitary stalk interruption syndrome. Horm Res. 2005;64: 266-273.

11. Bar C, Zadro C, Diene G, Oliver I, Pienkowski C, Jouret B, et al. Pituitary stalk interruption syndrome from infancy to adulthood: clinical, hormonal, and radiological assessment according to the initial presentation. PLoS One. 2015;10: e0142354.

12. Reiter EO, Price DA, Wilton P, Albertsson-Wikland K, Ranke MB. Effect of growth hormone (GH) treatment on the near-final height of 1258 patients with idiopathic GH deficiency: analysis of a large international database. J Clin Endocrinol Metab. 2006;91: 2047-2054.

13. Blethen SL, Compton P, Lippe BM, Rosenfeld RG, August GP, Johanson A. Factors predicting the response to growth hormone (GH) therapy in prepubertal children with GH deficiency. J Clin Endocrinol Metab. 1993;76: 574-579.

14. Greco M, Brugnara M, Zaffanello M, Taranta A, Pastore A, Emma F. Long-term outcome of nephropathic cystinosis: a 20-year single-center experience. Pediatr Nephrol. 2010;25: 2459-2467.

15. Markello TC, Bernardini IM, Gahl WA. Improved renal function in children with cystinosis treated with cysteamine. N Engl J Med. 1993;328: 1157-1162.

16. Brodin-Sartorius A, Tete MJ, Niaudet P, Antignac C, Guest G, Ottolenghi C, et al. Cysteamine therapy delays the progression of nephropathic cystinosis in late adolescents and adults. Kidney Int. 2012;81: 179-189.

17. Viltz L, Trauner DA. Effect of age at treatment on cognitive performance in patients with cystinosis. J Pediatr. 2013;163: 489-492.

18. Wuhl E, Trivelli A, Picca S, Litwin M, Peco-Antic A, Zurowska A, et al. Strict blood-pressure control and progression of renal failure in children. N Engl J Med. 2009;361: 1639-1650.

19. Wingen AM, Fabian-Bach C, Schaefer F, Mehls O. Randomised multicentre study of a low-protein diet on the progression of chronic renal failure in children. European Study Group of Nutritional Treatment of Chronic Renal Failure in Childhood. Lancet. 1997;349: 1117-1123.
