# Supplementary material for: Priority target conditions for algorithms for monitoring children's growth: Interdisciplinary consensus
Source: PLoS One. 2017 Apr 27;12(4):e0176464. doi: 10.1371/journal.pone.0176464 (PMC5407643; doi:10.1371/journal.pone.0176464)
Supplement: S5 Table — (DOC) [file pone.0176464.s005.doc]

**S5 Table.** Evidence supporting the presence of diagnostic criteria independent of auxological parameters for conditions selected as priority targets for children’s growth monitoring by algorithms**.**

| **Conditions** | **Diagnostic criteria** |
| --- | --- |
| **Celiac disease** | Histological analysis of duodenal biopsies or HLA-DQ2/HLA-DQ8 + serologies |
| **Crohn disease** | Macroscopic appearance during endoscopy, histological analysis of biopsies + biological and imaging findings |
|
| **Craniopharyngioma** | Histological analysis of biopsies/exeresis + imaging findings |
|
| **Turner syndrome** | Genotype 45X0 or mosaic |
| **Growth hormone deficiency with PSIS** | Biological (IGF-1 and growth hormone) and imaging findings (absence of a visible pituitary stalk, ectopic or absent posterior pituitary, and/or hypoplasia or aplasia of anterior pituitary) |
| **Infantile cystinosis** | Biochemical, ophthalmic, and genetic findings |
|
| **Juvenile nephronophthisis** | Histological analysis and genetic findings |
|
| **Hypothalamic-optochiasmatic astrocytoma** | Imaging findings ± histological analysis of biopsies/exeresis |
|
| PSIS: pituitary stalk interruption syndrome. | |

# **REFERENCES OF APPENDICES**

1. Husby S, Koletzko S, Korponay-Szabo IR, Mearin ML, Phillips A, Shamir R, et al. European Society for Pediatric Gastroenterology, Hepatology, and Nutrition guidelines for the diagnosis of coeliac disease. J Pediatr Gastroenterol Nutr. 2012;54: 136-160.

2. Levine A, Koletzko S, Turner D, Escher JC, Cucchiara S, de Ridder L, et al. ESPGHAN revised porto criteria for the diagnosis of inflammatory bowel disease in children and adolescents. J Pediatr Gastroenterol Nutr. 2014;58: 795-806.

3. Muller HL. Childhood craniopharyngioma. Recent advances in diagnosis, treatment and follow-up. Horm Res. 2008;69: 193-202.

4. Garnett MR, Puget S, Grill J, Sainte-Rose C. Craniopharyngioma. Orphanet J Rare Dis. 2007;2: 18.

5. Bondy CA. Care of girls and women with Turner syndrome: a guideline of the Turner Syndrome Study Group. J Clin Endocrinol Metab. 2007;92: 10-25.

6. Saenger P, Wikland KA, Conway GS, Davenport M, Gravholt CH, Hintz R, et al. Recommendations for the diagnosis and management of Turner syndrome. J Clin Endocrinol Metab. 2001;86: 3061-3069.

7. Argyropoulou M, Perignon F, Brauner R, Brunelle F. Magnetic resonance imaging in the diagnosis of growth hormone deficiency. J Pediatr. 1992;120: 886-891.

8. Arslanoglu I, Kutlu H, Isguven P, Tokus F, Isik K. Diagnostic value of pituitary MRI in differentiation of children with normal growth hormone secretion, isolated growth hormone deficiency and multiple pituitary hormone deficiency. J Pediatr Endocrinol Metab. 2001;14: 517-523.

9. Louvel M, Marcu M, Trivin C, Souberbielle JC, Brauner R. Diagnosis of growth hormone (GH) deficiency: comparison of pituitary stalk interruption syndrome and transient GH deficiency. BMC Pediatr. 2009;9: 29.

10. Elmonem MA, Veys K, Soliman N, Van Dyck M, Van Den Heuvel L, Levtchenko E. Cystinosis : a review. Orphanet Journal of Rare Diseases 2016.

11. Emma F, Nesterova G, Langman C, Labbe A, Cherqui S, Goodyer P, et al. Nephropathic cystinosis: an international consensus document. Nephrol Dial Transplant. 2014;29 Suppl 4: iv87-94.

12. Wilmer MJ, Schoeber JP, van den Heuvel LP, Levtchenko EN. Cystinosis: practical tools for diagnosis and treatment. Pediatr Nephrol. 2011;26: 205-215.

13. Wolf MT, Hildebrandt F. Nephronophthisis. Pediatr Nephrol. 2011;26: 181-194.

14. British Neuro-Oncology Society (2011) Guidelines on the diagnosis and management of Optic Pathway Glioma (OPG)

15. Poussaint TY, Barnes PD, Nichols K, Anthony DC, Cohen L, Tarbell NJ, et al. Diencephalic syndrome: clinical features and imaging findings. AJNR Am J Neuroradiol. 1997;18: 1499-1505.

16. Brauner R, Trivin C, Zerah M, Souberbielle JC, Doz F, Kalifa C, et al. Diencephalic syndrome due to hypothalamic tumor: a model of the relationship between weight and puberty onset. J Clin Endocrinol Metab. 2006;91: 2467-2473.
